# Enabling Access to Enhanced Care Models for Vulnerable Women

**DOI:** 10.55975/WNFJ1758

**Published:** 2025-09-01

**Authors:** Sally Haymes, Abbie McCleary, Raeesa Jassat, Naomi Charlton, Emma Meekin, Valerio Benedetto, James Hill

**Affiliations:** Liverpool Women’s Institute; Mersey Care NHS Foundation Trust; University of Central Lancashire; Alder Hey Children’s NHS Foundation Trust; University of Central Lancashire

## Abstract

Enhanced pregnancy care models for vulnerable women could increase utilisation of services and improve clinical outcomes for the mothers and their babies. This commentary critically appraised a systematic review which looked at the change in service utilisation and effectiveness of enhanced care for women in high-income countries, and found methodological limitations that should be considered when interpreting the review’s results. Expanding from the findings of the review, we drew implications for practice focused on the UK context as well as recommendations for future research.

## Introduction

Globally an estimated 287,000 women died during and following pregnancy and childbirth in 2020 globally.^1^ Women with vulnerabilities and complex social factors are at a higher risk of poorer outcomes, maternal death and stillbirth.^2^ They are less likely to engage early with maternity services and are at higher risk of developing complications, possibly due to their lack of antenatal care.^3^ Identifying and removing barriers can improve utilisation of maternity services, and may result in better clinical outcomes for women and their babies.^4^

The World Health Organization recommends commencing pregnancy care before 12-week gestation,^4^ a minimum of eight antenatal contacts within a midwife-led continuity-of-care model, and a minimum of four postnatal visits.^5^ These recommendations aim to reduce perinatal mortality, severe maternal morbidity and improve women’s experience of care. Furthermore, attendance of a skilled birth attendant is recognised as critical to improving maternal and newborn survival.^4–6^

However, lack of adherence to these recommendations puts women at risk of dying from pregnancy or birthing complications.^3^ Specific groups of women in high-income countries (HICs), such as refugees and migrants ^7^, those with disabilities ^8^, and adolescents^9^, disproportionately experience high rates of maternal mortality and severe maternal morbidity. Despite the availability of enhanced models of care in HICs, enablers and barriers to accessing these by vulnerable women, are not well understood^10^.

To address this gap, the systematic review by Bowden et al. (2023)^10^ aimed to determine whether care-seeking interventions increase utilisation of services in pregnancy, birthing, and the postnatal period (up to 6 weeks), as well as improving clinical outcomes, for women living with vulnerabilities, and their babies, in HICs. This commentary aims to critically appraise the review by Bowden et al. (2023)^10^ and expand upon the findings in the context of clinical practice.

## Methods

Bowden et al. (2023)^10^ searched 8 databases, including Medline and Cochrane Central, with the last search undertaken on 20^th^ June 2022. An additional backward citation search was undertaken. The inclusion criteria initially focused on randomised controlled trials (RCTs) only and was later expanded to include non-RCTs and cohort studies. All studies had to be set in HICs. The primary outcome was the utilisation of antenatal, intrapartum and postpartum healthcare services up to 6 weeks postpartum. Secondary outcomes included maternal mortality and severe maternal morbidity, infant mortality, birth outcomes (low birthweight <2.5 kg and preterm birth < 37 weeks), neonatal morbidity (APGAR score < 7 at five minutes, treatment with antibiotics, admission to neonatal intensive care unit, NICU), and neonatal respiratory events. No time limit was applied, while only studies written in English were considered.

Two reviewers independently conducted screening and assessment bias (RoB1 or ROBINS-i) with arbitration by a third reviewer. Data extraction was undertaken using a standard template. Initially the authors only performed meta-analysis on data from RCTs but then also included data from non-RCTs given the little heterogeneity between study designs and the expectation that the interaction between the effect of intervention and the choice of allocation was unlikely to impact on the results. Heterogeneity between study results was investigated using the I^2^ statistic, with sub-group analyses planned to explore any substantial heterogeneity. The GRADE framework was used to investigate the quality of the evidence underpinning the different outcomes.^11^

## Results

The review included nine studies (5729 women, age range: 12 to 45 years old). These studies were undertaken in United States (RCTs, n = 7) and Australia (Non-RCT and a prospective cohort study). Four studies focused on antenatal care, three studies on antenatal and postpartum care, one study on intrapartum and postpartum care, and the remaining study on postpartum care.

The 7 RCTs were predominantly assessed as having a low risk of bias concerning allocation concealment (n=5), attrition bias (n=4), and reporting bias (n=6). The remaining RCTs were classified as having an unclear risk of bias for these criteria. Conversely, a smaller number of studies had a low risk of bias for random sequence generation (n=2), performance bias (n=1) and detection bias (n=1), while the remaining studies were classified as having an unclear risk of bias. For the 2 non-RCTs, these studies were classified as having a low risk of bias (for bias in selection of participants, classification of interventions, deviations from intended interventions, missing data, measurement of outcomes, selection of the reported result) except for confounding variables where one study was classified to have a risk of bias.

In terms of the primary outcome of utilisation of maternal health services up to 6 weeks postpartum, the meta-analysis (RCTs only) found a statistically significantly higher odds of mothers attendance at antenatal classes (odds ratio (OR): 15.23; 95% confidence interval (CI): 10.73 to 21.61, I^2^: 62%, representing substantial heterogeneity) and at 6-8 week postpartum visit, (OR: 2.66; 95% CI: 1.94 to 3.64, I^2^: 0%) in the intervention group compared with the control group. The above results were reported to be associated with high certainty of evidence by the authors (according to the GRADE framework). Results from individual studies also indicated that the number of antenatal visits in the intervention group was statistically significantly higher than in the control group. No studies examined the utilisation of intrapartum care.

In terms of the infant-related secondary outcomes, in the meta-analysis the odds associated with low birth weight (<2500 g) (OR: 0.78; 95% CI: 0.64 to 0.95; p=0.01; I^2^: 0%), pre-term birth (<37 weeks) (OR: 0.68; 95% CI: 0.56 to 0.82; p<0.0001; I^2^: 19%, indicating that heterogeneity might not be important), and admission to NICU (OR: 0.80; 95% CI: 0.66 to 0.96; p=0.02; I^2^: 6%, indicating that heterogeneity might not be important) were statistically significantly smaller in the intervention group compared with the control group when combining RCTs with non-RCTs. However, when only including RCTs there was no evidence of statistically significant difference in the odds for low birth weight (OR: 0.90; 95% CI: 0.67 to 1.19; p=0.46), pre-term birth (OR: 0.84; 95% CI: 0.64 to 1.11; p=0.20), and admission to NICU (OR: 0.88; 95% CI: 0.69 to 1.13; p=0.33). Furthermore, no evidence of difference was observed in the meta-analysis for the risks of perinatal death and APGAR score < 7 at 5 minutes. The authors reported high certainty of evidence attached to all the above infant-related results. Maternal-related secondary outcomes were not analysed by the included studies, and no sub-group analysis was possible as appropriate data was not available.

## Commentary

### Critical appraisal of the review by Bowden et al. (2023)^10^

We critically appraised the review by Bowden et al. (2023)^10^ by using the AMSTAR-2 tool,^12^ and we found some issues that potentially impact on the overall quality of the study. First, with respect to the inclusion criteria, the authors set out to investigate the effectiveness of ‘enhanced care’ interventions, but this broad scope meant that there was a lack of specificity around the interventions considered. It was also unclear why the decision to include non-RCTs was made post hoc.

Second, the authors did not motivate their deviations from some of the protocol’s plans,^13^ in terms of expanding both the number of databases and the types of study designs taken into account (which ultimately included non-RCTs too).

Third, the formalisation of the search strategy did not include standard filters nor saw the involvement of an information specialist.

Fourth, while the screening process was conducted independently by two reviewers, it remained unclear whether the data extraction was also undertaken in duplicate.

Fifth, it was unclear in the protocol whether the meta-analysis was based on a fixed- or random-effects model (as both types were cited),^13^ and this clarification did not emerge from within the actual review either. Moreover, for some of the secondary outcomes, an unusual decision was made to include non-RCTs within the meta-analysis. Relevantly, when only RCTs were meta-analysed no statistically significant difference was found between intervention and comparator groups for the secondary outcomes.

Sixth, the risk of bias of the individual studies was not accompanied by a breakdown of grading, making it unclear to understand what it was based on. The methodological issues in the included RCTs would typically justify a downgrading in the certainty of the estimates. Additionally, there was substantial heterogeneity which would also justify downgrading the certainty of the evidence.

Lastly, no assessment of publication bias was undertaken by the authors as part of the meta-analysis.

### Implications for practice

It is difficult to say what impact the findings of Bowden et al. (2023)^10^ would have on the utilisation of maternal health services in the UK due to the varying natures of the interventions considered. The World Health Organization and the UK NHS recommend commencing pregnancy care before 12-week gestation in order to have a healthy pregnancy.^4,14^ However, low adherence to this recommendation calls for remedial actions such as providing more frequent and intensive antenatal appointments, encouraging partners to attend and act as a positive source of support, and promoting attendance through a gift incentive.^15^

Guidelines in the UK recommend 7 antenatal appointments for parous women and 10 antenatal appointments for nulliparous,^16^ without deviations for individuals with complex social factors.^17^ Despite the review finding that postnatal attendance at 6-8 weeks would increase with enhanced care interventions and provide an opportunity to discuss contraceptive options and the vaccination pathway of the newborn,^18^ currently in the UK a 6-8 week postpartum contact is not offered within midwifery services.

A recent study of women with complex social factors, not included in the review by Bowden et al. (2023),^10^ identified that increasing antenatal appointments above the recommended amount could reduce the stillbirth rate for this cohort of women.^19^ Moreover, evidence suggests that one-on-one care, group antenatal care, attendance of skilled care at birthing and clear health messages received through a prenatal partner during pregnancy, labour and the postpartum period have the potential to reduce maternal morbidity and mortality.^10^

By tailoring pregnancy and birthing care towards individual needs, women, healthcare providers and support people will be able to request resources in the perinatal period and, via a preventive intervention (e.g. health education), in the preconception period.^20^ Mental wellbeing should also be considered at each stage of care, from labour, birth, lactation, up to the 12-week postpartum visit,^10^ focusing on sources of trauma for women, such as giving birth and attempting to breastfeed.

### Recommendations for future research

The review by Bowden et al. (2023)^10^ demonstrates the lack of UK-based research to inform the planning and delivery of multi-agency antenatal education and services. Therefore, future research could aim to:

1. Clarify the definition of vulnerabilities and complex social factors to carry out research focusing on specific priority groups (e.g. women affected by intimate partner violence or domestic abuse, involved with safeguarding services, under the care of secondary mental health services);
2. Identify good practice in UK maternity teams, including reviewing guidance that could inform future research;^17^
3. Establish what an ‘enhanced care’ should look like for women with vulnerabilities and complex social factors in terms of numbers of appointments and evidence-based content, considering the benefit of continuity of care;^21^
4. Determine how to improve access, uptake and engagement to ‘enhanced care’;
5. Compare process and clinical outcomes from delivering enhanced services in specialist sites versus delivering non-enhanced services.

## Conclusions

This commentary critically appraised the systematic review by Bowden et al. (2023)^10^ which looked at the effects of enhanced pregnancy care in service utilisation and clinical outcomes for vulnerable women and their babies. Methodological limitations particularly around the inclusion of RCTs and non-RCTs and potential downgrading of the certainty of evidence should be considered when interpreting the results. We further discussed how enhanced care could be implemented within UK pregnancy care and delineated evidence gaps to be addressed by future research.
